# Mutations in the C-terminal region affect subcellular localization of crucian carp herpesvirus (CaHV) GPCR

**DOI:** 10.1007/s11262-016-1325-y

**Published:** 2016-04-08

**Authors:** Jun Wang, Lang Gui, Zong-Yan Chen, Qi-Ya Zhang

**Affiliations:** State Key Laboratory of Freshwater Ecology and Biotechnology, Institute of Hydrobiology, Chinese Academy of Sciences, Graduate University of Chinese Academy of Sciences, Wuhan, 430072 China; Fisheries and Life Science, Shanghai Ocean University, Shanghai, 200120 China

**Keywords:** G protein-coupled receptor (GPCR), Crucian carp herpesvirus (CaHV), C-terminal, Subcellular localization, *N*-myristoylation site

## Abstract

G protein-coupled receptors (GPCRs) are known as seven transmembrane domain receptors and consequently can mediate diverse biological functions via regulation of their subcellular localization. Crucian carp herpesvirus (CaHV) was recently isolated from infected fish with acute gill hemorrhage. CaHV GPCR of 349 amino acids (aa) was identified based on amino acid identity. A series of variants with truncation/deletion/substitution mutation in the C-terminal (aa 315–349) were constructed and expressed in fathead minnow (FHM) cells. The roles of three key C-terminal regions in subcellular localization of CaHV GPCR were determined. Lysine-315 (K-315) directed the aggregation of the protein preferentially at the nuclear side. Predicted *N*-myristoylation site (GGGWTR, aa 335–340) was responsible for punctate distribution in periplasm or throughout the cytoplasm. Predicted phosphorylation site (SSR, aa 327–329) and GGGWTR together determined the punctate distribution in cytoplasm. Detection of organelles localization by specific markers showed that the protein retaining K-315 colocalized with the Golgi apparatus. These experiments provided first evidence that different mutations of CaHV GPCR C-terminals have different affects on the subcellular localization of fish herpesvirus-encoded GPCRs. The study provided valuable information and new insights into the precise interactions between herpesvirus and fish cells, and could also provide useful targets for antiviral agents in aquaculture.

## Introduction

The G protein-coupled receptor (GPCR) family consists of the largest and most versatile group of cell surface receptors which shares a common topology of seven transmembrane domains. Most GPCRs have a common molecular architecture: an extracellular N-terminal segment and a cytoplasmic C-terminal tail containing important regions which are critical for ligand-dependent signaling and receptor trafficking [1]. GPCRs are intricately involved in a diverse array of physiological and pathophysiological processes [2]. The structure and function of mammals or mammalian virus-encoded GPCRs has been well studied [3]. Changes in GPCR C-terminal structures may verify their functional location through colocalization studies [4]. GPCRs can help the virus survive host immune attacks and contribute to immune evasion by targeting the cellular MHC I class for degradation [5].

Aquaculture has rapidly developed and it has been believed to become a true production industry because an artificial propagation technique has been successfully established [6]. But aquaculture is plagued with viral disease problems [7, 8]. In recent years, there has been virulent, massive mortality, usually due to acute gill hemorrhagic disease of cultured carp caused by herpesvirus [9, 10]. The fish herpesvirus infection and herpesvirus disease became widely reported in the world [11, 12]. Although genome sequence data demonstrate that the fish virus genome contains the gene encoding a GPCR, little is known about the role of GPCR in fish viral disease [13].

A key annotation is the prediction of a protein’s subcellular localization and the subcellular localization is an essential functional characteristic of proteins. A challenging task of the virus–host interactions in aquaculture animals is the identification and localization of viral proteins to determine the molecular mechanisms of action [14]. In the present study, a highly pathogenic herpesvirus (CaHV) was isolated from diseased crucian carp with acute gill hemorrhage, and the viral genome encodes a GPCR. On this basis, a series of truncations, deletions, and substitution expression plasmids for CaHV GPCR C-terminal-enhanced green fluorescent protein (EGFP) fusion were generated. This system served as CaHV GPCR key region analysis, which helped to provide insight into its intracellular localization and colocalization with the host organelle.

## Materials and methods

### Virus isolation and viral genomic DNA extraction

Diseased crucian carp (*Carassius auratus*), which showed apparent hemorrhage on the gills and external surface of the fish. The virus (CaHV) isolation was performed by methods as described previously [15, 16]. Purified virus particles were negatively stained with 2 % (*w/v*) phosphotungstic acid and then examined with a JEM-1230 electron microscope (JEOL, Tokyo, Japan). CaHV genome DNA was extracted from purified virus particles using an E.Z.N.A Viral DNA Kit (OMEGA, VT, USA), following the manufacturer’ s protocols and used for CaHV GPCR cloning.

### Cloning, sequencing, and analysis of CaHV GPCR gene

CaHV genome DNA was used as template to amplify the GPCR with a pair of GPCR-F/R primers (Table 1). The PCR reaction was performed in a volume of 25 μL, containing 0.5 U of Taq polymerase (TransGen, Beijing, China), 1 × Taq buffer, 0.2 μM of each primer, 0.2 mMd NTPs, and 1 μL DNA. PCR conditions were carried out as follows: pre-denaturation at 94 °C for 5 min; 30 cycles of denaturation at 94 °C for 30 s, annealing at 55 °C for 30 s, and extension at 72 °C for 1 min; followed by a final extension step of 72 °C for 10 min. The PCR product was analyzed by electrophoresis in 1 % agarose gel and visualized under a gel imaging analysis system (Syngene G:Box) after staining with ethidium bromide (EB).

**Table 1:** Primers used in this study (enzyme cleavage site is underlined)

Primer name	Primer sequence (5′–3′)
GPCR-F	AGATGTTCAAGTTGTTGCTG
GPCR-R	CAACAACATTGTGAAAAACA
GPCR-GFP-F	AAAGAATTCGATGTTCAAGTTGTTGCTGCTG (EcoRI)
GPCR-GFP-R	AAAGGATCCTACAGACTCTTCTTCTTCGTCG (BamHI)
GPCR316-349-R	CTCGGATCCCTTCCACAATAGAAAACAAGGGT (BamHI)
GPCR315-349-R	GGTGGATCCCCACAATAGAAAACAAGGGTTCA (BamHI)
GPCR-D315-F	CTATCAAAGTACTCGGTACCCCACAATAGAAAACAAGGGT
GPCR-D315-R	ACCCTTGTTTTCTATTGTGGGGTACCGAGTACTTTGATAG
GPCR-K315A-F	TGTTTTCTATTGTGGGCAGGTACCGAGTACTT
GPCR-K315A-R	AAGTACTCGGTACCTGCCCACAATAGAAAACA
GPCR-D315-326-F	TGTTTTCTATTGTGGTCATCCAGACTGATG
GPCR-D315-326-R	CATCAGTCTGGATGACCACAATAGAAAACA
GPCR-D315-329-F	TGTTTTCTATTGTGGCTGATGAGCTGTTT
GPCR-D315-329-R	AAACAGCTCATCAGCCACAATAGAAAACA
GPCR-D315-334-F	TGTTTTCTATTGTGGGGTGGCGGCTGGA
GPCR-D315-334-R	TCCAGCCGCCACCCCACAATAGAAAACA
GPCR-D315-340-F	TGTTTTCTATTGTGGATGATCGACGAAGA
GPCR-D315-340-R	TCTTCGTCGATCATCCACAATAGAAAACA
GPCR-D327-329-F	AGACGTACTGGATTCCTGATGAGCTGTTT
GPCR-D327-329-R	AAACAGCTCATCAGGAATCCAGTACGTCT
GPCR-D335-340-F	CTGATGAGCTGTTTTATGATCGACGAAG
GPCR-D335-340-R	CTTCGTCGATCATAAAACAGCTCATCAG
Plasmid-R	TGTTTCAGGTTCAGGGGGAGGTGTG

The target fragment was purified using a Silica Bead DNA Gel Extraction Kit (Fermentas, MA, USA) according to the manufacturer’s protocol, cloned into pMD-18T vector (TaKaRa, Tokyo, Japan), and sequenced in both directions using an automated ABI sequencer (Sangon, Shanghai, China).

The nucleotide and deduced amino acid (aa) sequences were analyzed using the EditSeq program (Lasergene). Homology searches of nucleic acid and protein databases were performed using BLAST at the National Center for Biotechnology Information server. Transmembrane domains (TMs) were predicted using TMHMM 2.0 (http://www.cbs.dtu.dk/services/TMHMM-2.0) and TMpred (http://www.ch.embnet.org/software/TMPRED_form.html). A two-dimensional model of the CaHV GPCR was created with TOPO2 software (http://www.sacs.ucsf.edu/TOPO2/). A region scan program was used to predict functional domains within the C-terminal of the CaHV GPCR [17].

### Plasmid construction

The full-length CaHV GPCR (aa 315–349) was amplified from CaHV genome DNA, digested with EcoRI/BamHI and inserted into pEGFP-N3 (Clontech, CA, USA) to give the plasmid pEGFP-GPCR. Additionally, a series of CaHV GPCR expression plasmids containing C-terminal truncations, deletions, or substations were constructed to identify the key regions responsible for subcellular localization. At the same time, the plasmid pEGFP-empty (pEGFP-N3) was used as negative control.

The stop codon and restriction sites were introduced at aa 315 and 316 of the C-terminal of the GPCR by PCR amplification using plasmid pEGFP-GPCR as a template. Each PCR product was digested with EcoRI/BamHI and then ligated to pEGFP-N3, which had been digested with the same enzymes, yielding plasmids pEGFP-GPCR_T-316–349_ and pEGFP-GPCR_T-315–349_ carrying C-terminal truncations.

An overlap extension PCR was employed, which included two rounds of PCR. Briefly, two DNA fragments were obtained from the first round PCR with the corresponding primers. They were then used as template for the second round PCR. Then, the DNA fragments with the corresponding region deleted were obtained. These DNA fragments were digested with EcoRI/BamHI and ligated to pEGFP-N3 plasmid with the same enzyme treatment. Using this PCR strategy, aa 315–326, 315–329, 315–334, and 315–340 within the C-terminal of CaHV GPCR were successively deleted, resulting in the plasmids pEGFP-GPCR_D-315–326_, pEGFP-GPCR_D-315–329_, pEGFP-GPCR_D-315–334_, and pEGFP-GPCR_D-315–340_, respectively. Additionally, pEGFP-GPCR_D-K_ was constructed with the lysine (K-315) deleted. Plasmid pEGFP-GPCR_D-KSSR_was constructed with the K and SSR (aa 315 and aa 327–329) deleted. Plasmid pEGFP-GPCR_D-KGGGWTR_was also constructed, with the K and GGGWTR (aa 315 and aa 335–340) deleted.

To generate plasmid pEGFP-GPCR_315-K/A_, primers GPCR-315 K/A-F/R that contained mutations with the lysine residue (K-315) substituted by alanine (A-315) were used with the same overlap extension PCR method as described above. Primers, enzymes, and the resulted plasmids are mentioned in Table 1. All constructed plasmids mentioned above were confirmed by restriction enzymes and DNA sequencing.

### Transfection of plasmids and fluorescent observation

Fathead minnow (FHM) cells were maintained at 25 °C in a TC199 medium supplemented with 10 % fetal bovine serum (FBS). Before transfection, cells were grown to 90 % confluence on a microscopic coverslip in 6-well plates and then transfected with the lipofectamine 2000 (Invitrogen, CA, USA) reagent (according to the manufacturer’s instructions) plus 2 μg plasmid DNA. At 24 h after transfection (h p.t.), cells were fixed with 4 % paraformaldehyde (PFA) in PBS for 30 min and stained with Hoechst 33,342 (Sigma, MO, USA) for 15 min. The cells were visualized under a Leica DM IRB fluorescence microscope (objective 100×). Images were processed using the Adobe Photoshop program. Green fluorescence displayed the distribution of target protein, and the cell nucleus was indicated by blue fluorescence [18].

To investigate the relationship between CaHV GPCR and cell organelles such as endoplasmic reticulum (ER), mitochondria (MT), and Golgi apparatus (Golgi), three organelle-specific markers including plasmid pDsRed2-ER, pDsRed2-MT, and pDsRed2-Golgi (Clontech, CA, USA) were used. The full-length or truncated CaHV GPCR expression plasmids were co-transfected with pDsRed2-ER, pDsRed2-MTor, and pDsRed2-Golgi; and subjected to fluorescence observation at 24 h p.t. as described above. Green fluorescence displayed the distribution of target protein and red fluorescence indicated the distribution of organelles. Yellow fluorescence showed that the proteins were well colocalized.

## Results

### The typical symptoms of diseased crucian carp and morphology of the virus particle

**Fig. 1:**
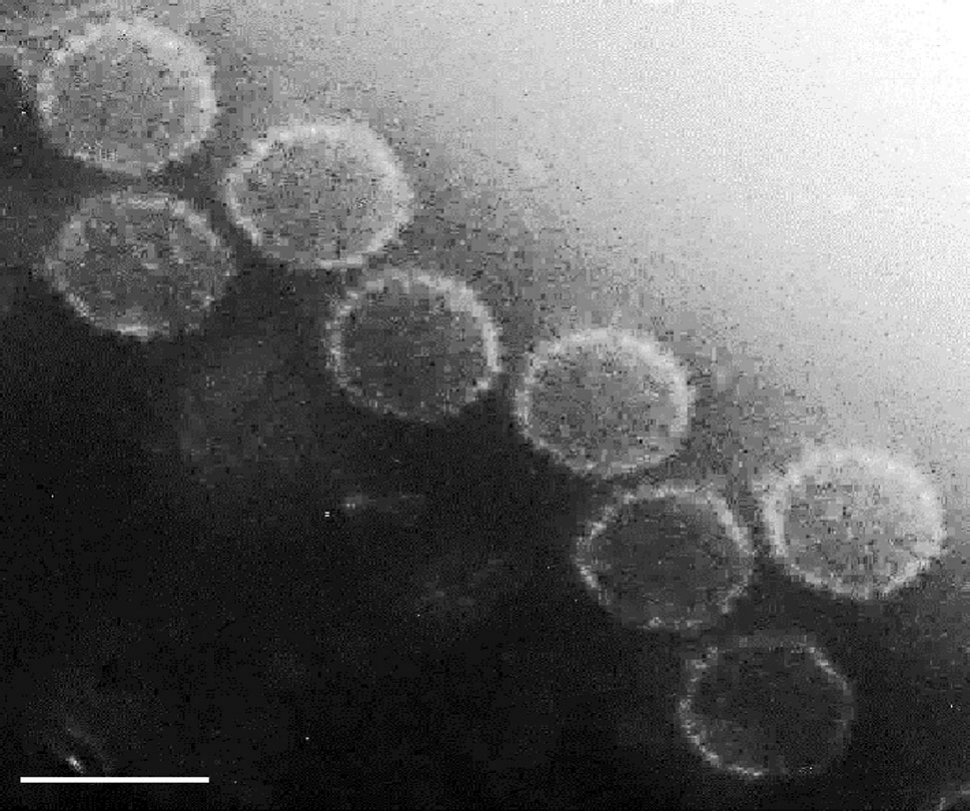
Electron micrograph of CaHV. Purified CaHV was negatively stained. The virus particles had icosahedral capsids with protrusions on the surface and had an estimated diameter of 97 nm. *Bar* = 100 nm

Diseased crucian carp shows acute systemic bleeding, especially in gills, which could result in extremely high mortality rates. Signs were also seen on petechial hemorrhages at fin bases and skin. The virus was isolated and purified by differential centrifugation and sucrose density gradient centrifugation from the tissue homogenates of diseased fish as described previously [15, 16]. Electron microscopic observation showed these virus particles had icosahedral capsids with protrusions approximately 97 nm in diameter (Fig. 1). These particles exhibit typical herpesvirus morphology, and therefore are referred to as CaHV.

### CaHV GPCR sequences, the secondary structure and the key regions

**Fig. 2:**
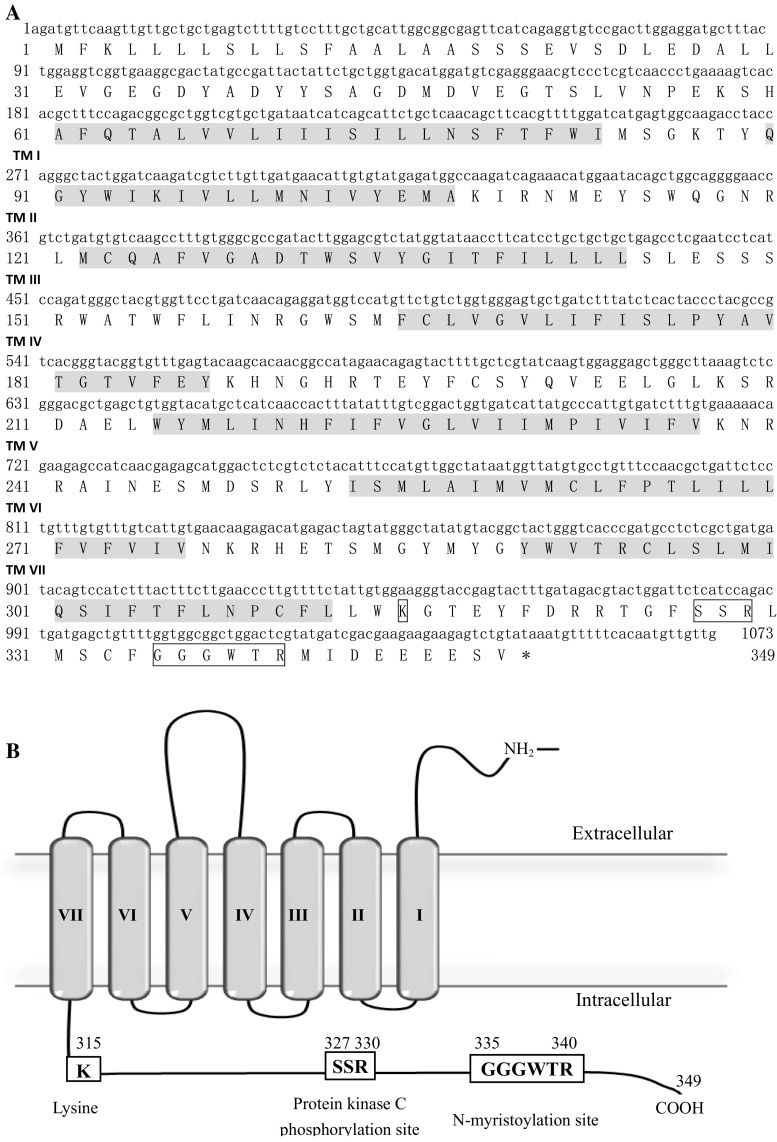
Primary sequence, secondary structure, and C-terminal tails of CaHV GPCR. **a** Nucleotide and amino acid sequence of CaHV GPCR. The putative seven transmembrane domains (I–VII) are *shaded* and three regions are shown as *boxes*. **b** The secondary structure of CaHV GPCR showing the seven membrane spanning domains (I–VII), connecting loops and C-terminal tail with the three key regions. A lysine (K-315, at the position 315), protein kinase C phosphorylation site (PKCs, three residues SSR or Ser-Ser-Arg, at the positions 327–329), and *N*-myristoylation site (six residues GGGWTR or Gly-Gly-Gly-Trp-Thr-Arg, at the positions 335–340) are shown with *boxes*, respectively

The genome DNA was obtained from the purified CaHV and the template was used for the amplification of GPCR with the designed primers (Table 1). A 1073 bp DNA fragment was amplified, and the sequence analysis showed the fragment containing a complete open reading frame (ORF), which was 1050 bp in length and encoding 349 aa (Fig. 2a). Transmembrane prediction indicated that CaHV GPCR had a central core domain with seven transmembrane regions (I–VII), including four extracellular domains and four cytoplasmic domains. The structures shared all the characteristics of other GPCRs (Fig. 2b). The C-terminal of the CaHV GPCR contained three key regions: a lysine residue (K-315, aa 315), a predicted protein kinase C (PKC) phosphorylation site (SSR or Ser-Ser-Arg, three residues at the positions 327–329), and a predicted N-myristoylation site (GGGWTR or Gly-Gly-Gly-Trp-Thr-Arg, six residues at the positions 335–340); which were predicted to act as subcellular localization, as shown in Fig. 2b.

### The role of three key regions in subcellular localization of CaHV GPCR

**Fig. 3:**
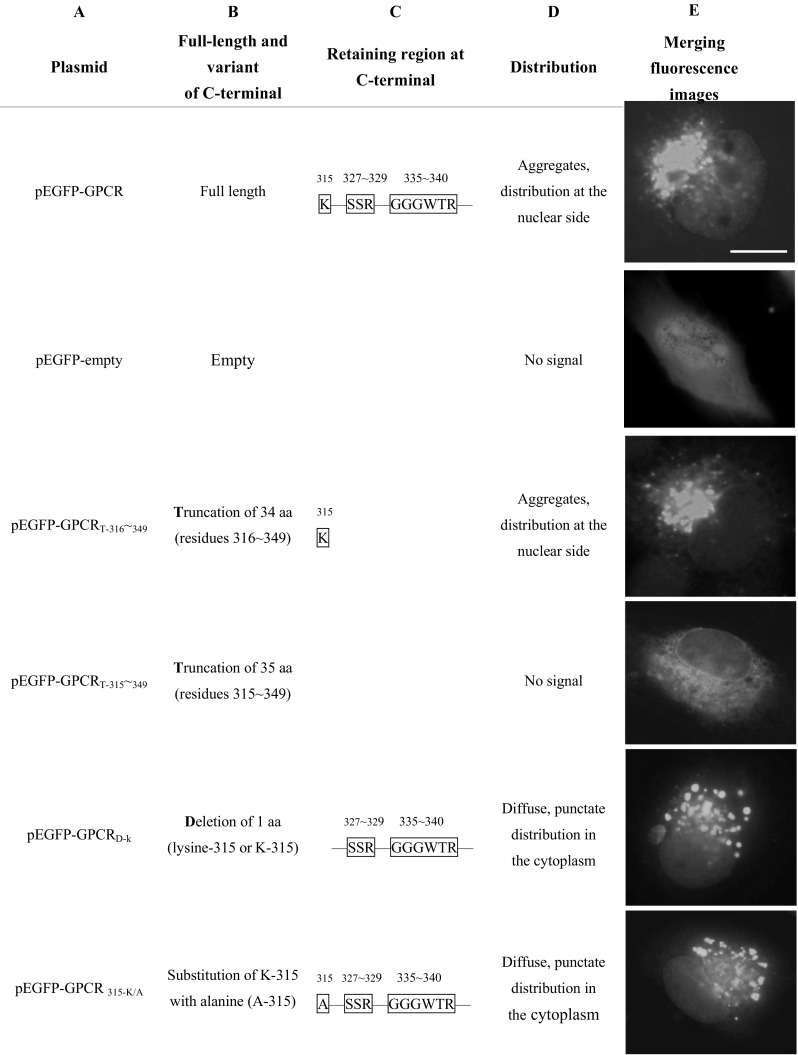

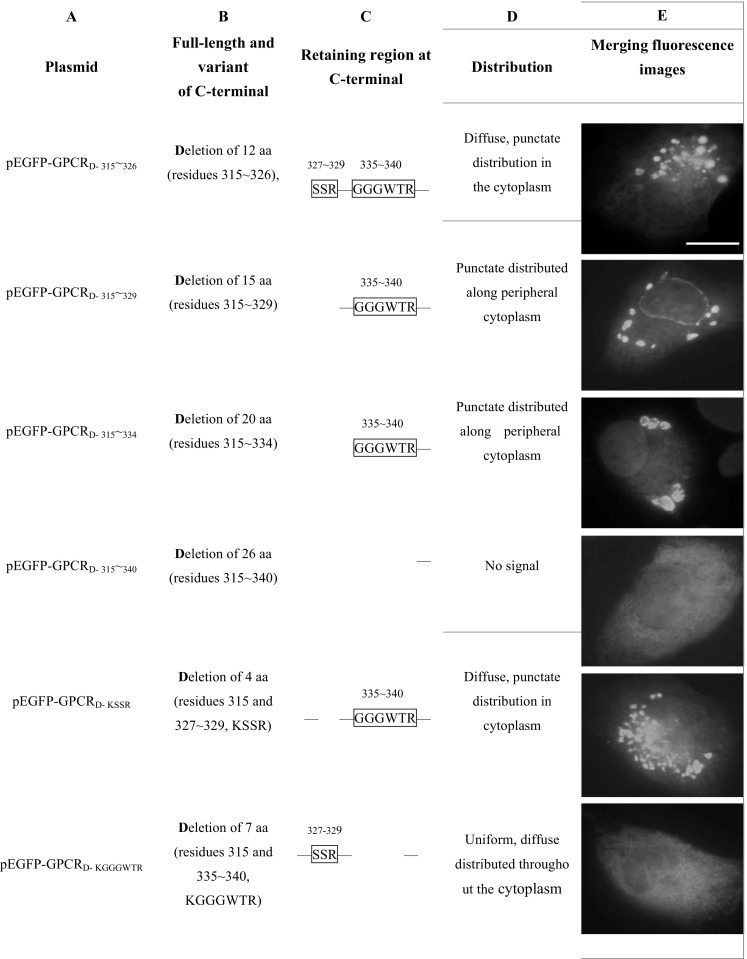
Three key regions of CaHV GPCR play important role in the subcellular localization of GPCR. FHM cells were transfected with expression plasmid pEGFP-GPCR or pEGFP-empty control. At 24 h p.t., cells were fixed for fluorescence observation. Plasmids corresponding to different lengths of CaHV GPCR C-terminal EGFP fusion proteins. Row **a**. Plasmid names, the plasmids expressing full-length, truncations (T), deletions (D), or substitutions (/) of GPCR C-terminal. The numbers at the plasmid *bottom right* indicate the position of the C-terminal residues. Row **b**. Altered aa on number and site (the *numbers* indicate aa residues). Row **c** The retaining region (inside the *box*) present at the C-terminal (the *numbers* indicate aa residues). Row **d** The distribution of expressed products in fish FHM cells. Row **e** The merging fluorescence images of EGFP reporter plasmid expression and subcellular distribution. *Bar* = 5 μm. The result came from three independent experiments, and at least ten cells were viewed at each time

FHM cells were transfected with expression plasmid pEGFP-GPCR containing an intact C terminus of a CaHV GPCR. At 24 h p.t., FHM cells were fixed for fluorescence observation. Merging fluorescence images showed that the variably sized fluorescence had a tendency to aggregate and was located at the nuclear side of the cell. pEGFP-GPCR_T-316–349_ with C-terminal 34 aa (residues 316–349) truncated, containing lysine residues (lysine-315 or K-315), displayed similar distribution to the full-length CaHV GPCR. However, pEGFP-GPCR_T-315–349_ with C-terminal 35 aa (residues 315–349) truncated, pEGFP-GPCR_D-k_ with C-terminal 1 aa (K-315) deleted, and pEGFP-GPCR_315-K/A_ with C-terminal containing alanine (A-315) instead of K-315 were found to have distributions with obvious changes. They exhibited fluorescent labeling diffusely distributed in the cytoplasm rather than aggregated at the nuclear side (Fig. 3). These data revealed that K-315 in C-terminal was a key region and it could direct CaHV GPCR aggregation and preferential localization at the nuclear side.

Furthermore, all the expression plasmids containing GGGWTR region (residues 335–340), including pEGFP-GPCR_D-k_, pEGFP-GPCR _315-K/A_, pEGFP-GPCR_D-315–326_ with C-terminal 12 aa (residues 315–326) deleted, pEGFP-GPCR_D-315–329_ with C-terminal 15 aa (residues 315–329) deleted, pEGFP-GPCR_D- 315–334_ with C-terminal 20 aa (residues 315–334) deleted, and pEGFP-GPCR_D-KSSR_ with C-terminal 4 aa (residues 315 and 327–329, KSSR) deleted had a punctate distribution on the periplasmic side and/or cytoplasm. However, punctate distribution disappeared in all the expression plasmids missing GGGWTR region, such as pEGFP-GPCR_T-315–349_, pEGFP-GPCR_D-315–340_ with C-terminal 26 aa (residues 315–340) deleted and pEGFP-GPCR_D-KGGGWTR_ with 7 aa (residues 315 and 335–340, KGGGWTR) deleted. They became a finely granular fluorescence with uniform distribution (Fig. 3). The region GGGWTR was found responsible for CaHV GPCR punctate distribution.

Expression plasmid pEGFP-GPCR_D-KGGGWTR_ with SSR region (aa 327–329) was then selected to test the effect of SSR on CaHV GPCR subcellular distribution, and it did not exhibit specific signal in the cytoplasm. The results turned out the same in expression plasmids pEGFP-GPCR_T-315–349_, pEGFP-GPCR_D-315–340_, and the control plasmid pEGFP-empty, in which C-terminals were missing K-315, GGGWTR, and SSR. However, if the two regions, SSR and GGGWTR (aa 334–340) both remained, such as in pEGFP-GPCR_D-k_, pEGFP-GPCR_315-K/A_, and pEGFP-GPCR_D-315–326_, CaHV GPCR were still able to have punctate distribution exhibited in cytoplasm, rather than a finely granular, uniformly distributed green fluorescence (Fig. 3). The results indicated that SSR and GGGWTR together determined the punctate distribution in cytoplasm.

### K-315 is responsible for CaHV GPCR colocalization with Golgi apparatus

**Fig. 4:**
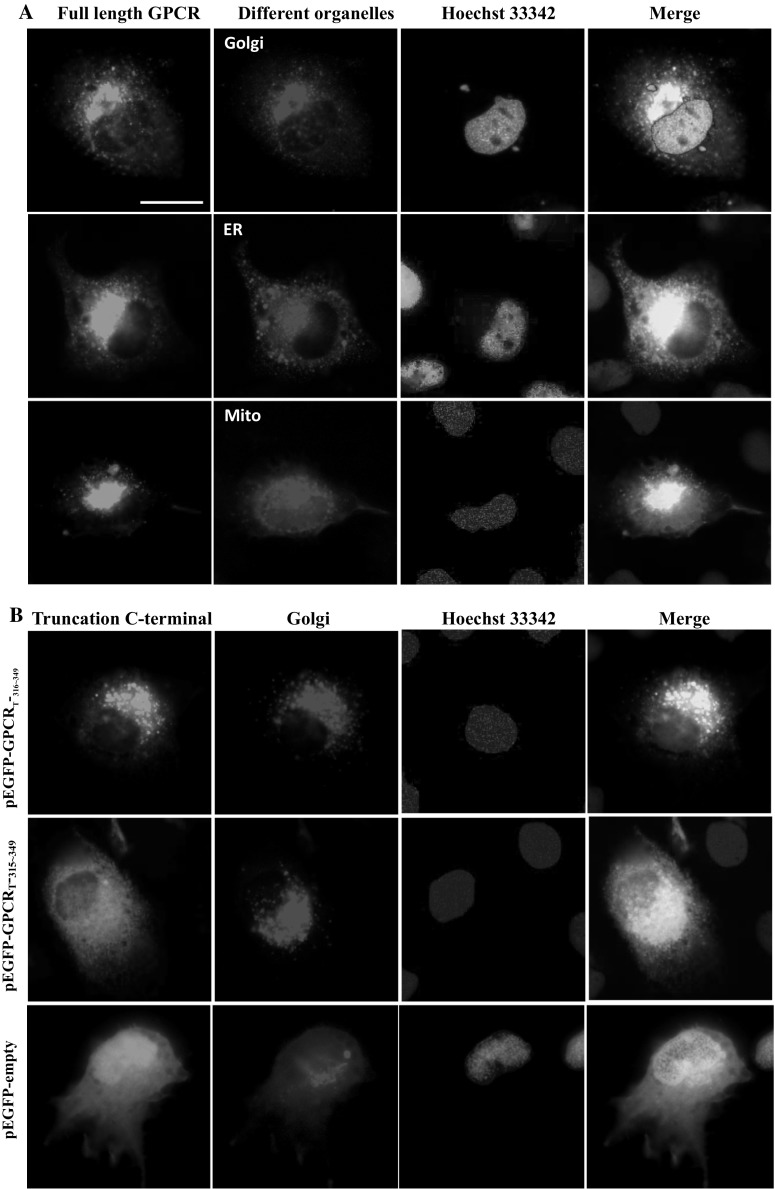
Colocalization of different length GPCR C-terminal with organelle-specific markers. FHM cells were co-transfected organelle-specific marker plasmid with expression plasmid pEGFP-GPCR or pEGFP-empty control. At 24 h p.t., cells were fixed for fluorescence observation. **a** Colocalization was seen between full-length GPCR and Golgi apparatus (Golgi), and did not colocalize with mitochondria (Mit) or endoplasmic reticulum (ER). **b** GPCR_T**-**316–349_ containing lysine-315 (K-315) colocalized with Golgi, but GPCR_T**-**315–349_ without K-315 or empty control did not colocalize with Golgi. The result came from three independent experiments, and at least ten cells were viewed at each time

The expression plasmid pEGFP-GPCR, containing full-length CaHV GPCR, was co-transfected with different organelle marker plasmids including pDsRed2-ER, pDsRed2-MT and pDsRed2-Golgi, respectively. At 24 h p.t., FHM cells were fixed for fluorescence observation. Merged images of green (CaHV GPCR) and red (organelle marker) signals showed good colocalization (yellow as isosurface) between the CaHV GPCR and the Golgi apparatus, but not with the endoplasmic reticulum or mitochondria (Fig. 4a).

The truncations of C-terminals, including pEGFP-GPCR_T-316–349_ and pEGFP-GPCR_T-315–349_, and the control plasmid pEGFP-empty were co-transfected with pDsRed2-Golgi, respectively. Merged images of green (the truncations) and red (Golgi apparatus marker) signals showed good colocalization (yellow as isosurface) between pEGFP-GPCR_T-316–349_ and the Golgi apparatus, but not with pEGFP-GPCR_T-315–349_ (K-315 missing) or pEGFP-empty (Fig. 4b). The results confirmed and extend that K-315 in the C-terminal is required for CaHV GPCR colocalization with the organelle.

### Schematic representation of the effects of mutations on subcellular localization

**Fig. 5:**
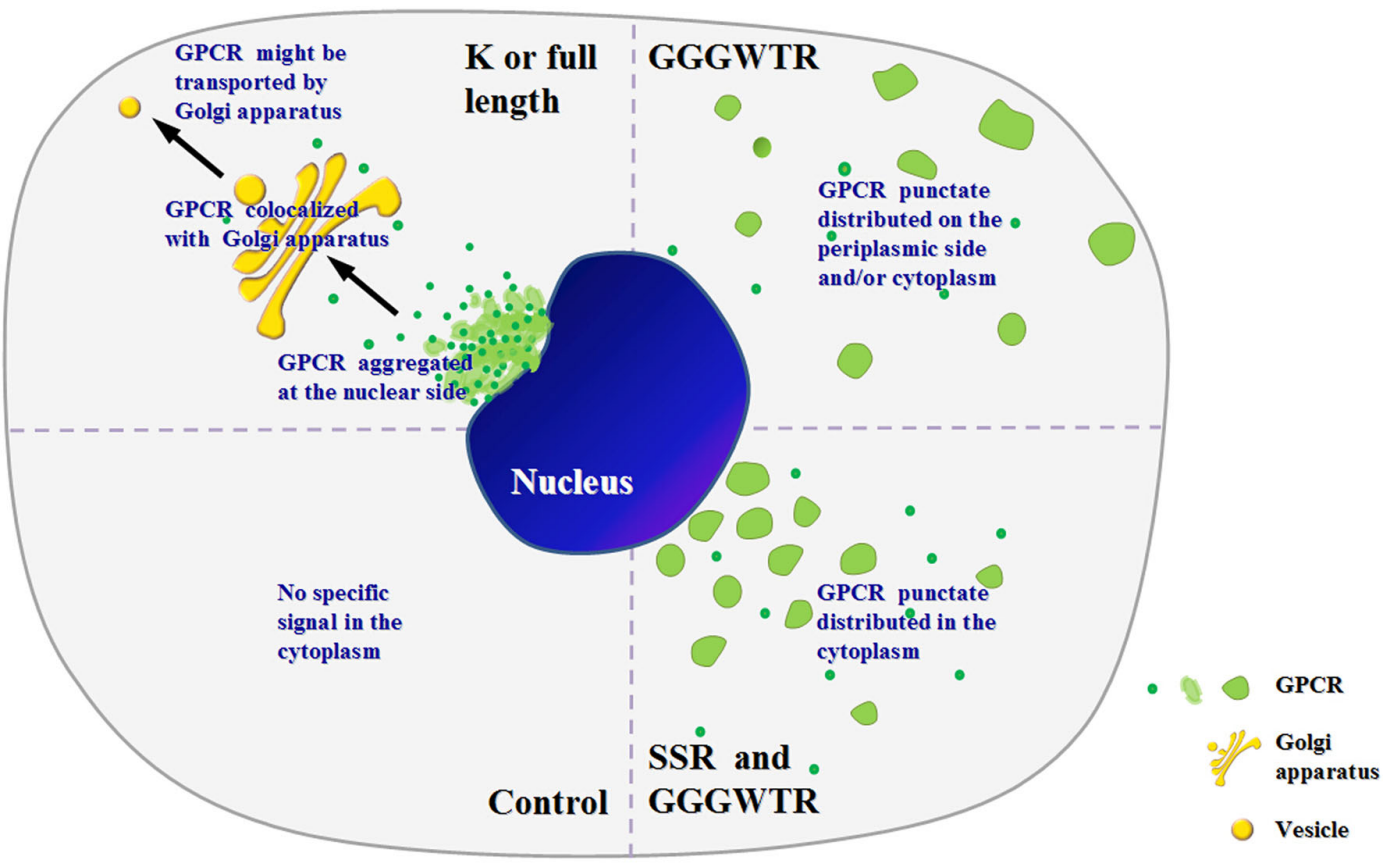
Schematic summary of the effects of C-terminal mutants on the subcellular localization of CaHV GPCR. The variants are represented by four circumstances (*dotted line*), including K or full length, GGGWTR, SSR and GGGWTR, and control. K or full length: When region K-315 or the intact C-terminal was retained, CaHV GPCR formed aggregates at the nuclear side, and colocalized with the Golgi apparatus and might be transported by Golgi apparatus. GGGWTR: when region GGGWTR was retained but regions K-315 and SSR were missing on the C-terminal, CaHV GPCR showed punctate distribution on the periplasmic side and/or cytoplasm. SSR and GGGWTR: when regions SSR and GGGWTR were both retained, CaHV GPCR exhibited a punctate distribution in the cytoplasm. Control: When empty control or the C-terminal excluding K-315 and GGGWTR were presented, no specific signal was exhibited in the cytoplasm. The distributions of the variants are represented by *green* (diffusion, uniform, or punctate). CaHV GPCR colocalizing with Golgi apparatus is represented by *yellow*. The *arrows* indicate CaHV GPCR and associated proteins might intracellularly transport to peripheral cytoplasm by Golgi apparatus

Subcellular localizations of CaHV GPCR have been intensively studied for different mutations of C-terminal. Figure 5 is a schematic summary of the key regions and their possible roles. When K-315 or the intact C-terminal was retained (K or full length), CaHV GPCR formed aggregates at the nuclear side, and colocalized with the Golgi apparatus. But when K-315 and SSR regions were missing on the C-terminal, CaHV GPCR showed punctate distribution only when the GGGWTR region (GGGWTR) was retained. When SSR and GGGWTR regions were retained (SSR and GGGWTR), CaHV GPCR exhibited a punctate distribution in the cytoplasm. When empty control or the C-terminal excluding K-315 and GGGWTR were presented (control), no specific signal was exhibited in the cytoplasm.

Analysis showed that CaHV GPCRC terminal was very dynamic, might be mainly involved in transporting the virus via the Golgi apparatus. Since full-length CaHV GPCR C-terminal localizations, and their variants, were not confined to the nuclear side or periplasm, they made for an interesting study of CaHV interaction with the host, as did the CaHV GPCR’s exhibition of its intracellular activity.

## Discussion

Subcellular localization is essential to protein function. Identification of key regions allows for the prediction of protein subcellular localization and function. Changes in the GPCR’s subcellular localization and functioning may cause diseases [19, 20]. The characterization and localization of regions is a fundamental approach to a better understanding of possible function and evolutionary relationships of the corresponding GPCRs. Several research teams have reported that viral GPCRs are highly significant for viral replication and for virus-induced pathogenesis in the hosts [21]. The signaling and trafficking properties of GPCRs are often highly malleable [22]. Some GPCR regions are known to be highly conserved, whereas the others represent potential novel regions. It is reported that lysine acetylation is a reversible post-translational modification that plays a crucial role in subcellular location, regulating protein function, chromatin structure, and gene expression [23, 24]. Although the mechanism of effects by lysine residue was not fully understood, there is evidence that it is important for directing the trafficking of virus between the nucleus and cytoplasm [25].

Several regions on C-terminal were involved in subcellular localization of CaHV GPCR, afforded by fluorescence-based analysis. We uncovered that a role that lysine-315 plays in CaHV GPCRs is that it can regulate subcellular localization, protein aggregation at nuclear side, and can also control colocalization closely with the Golgi apparatus. Therefore, it was clear that the role played by lysine residue in C-terminal of CaHV GPCR is irreplaceable, and it could be hypothesized that GPCR might be involved mainly in progeny particles of fish herpesvirus transporting through the Golgi apparatus from the nucleus, the position of replication, to the cell periphery. The subcellular localization of GPCR might involve CaHV assembly as the Golgi apparatus is the assembly site for a number of complex enveloped viruses [26]. The SSR region and GGGWTR region is believed to function as a protein kinase C (PKC) phosphorylation site and *N*-myristoylation site, respectively. Phosphorylation plays critical roles in the regulation of the signal transduction and life activities within the cell [27]. There is now considerable evidence that the kinases play important roles in viral infection and lifecycles [28, 29]. Whereas *N*-myristoylation is the attachment of myristic acid, a 14-carbon saturated fatty acid [30]. Post-translational modification of *N*-myristoylation provides an important approach for regulating protein subcellular localization, stability, trafficking, aggregation, interaction with effectors and other aspects of protein function [31]. But the role of protein kinase C (PKC) phosphorylation site and *N*-myristoylation site subcellular localization in the functions of fish herpesvirus is just beginning to receive attention. By integrating a fluorescence-based analysis system with bioinformatics techniques, we have clarified the key regions and the intracellular localization of GPCR from herpesvirus (CaHV) which caused hemorrhagic gill disease in crucian carp. To the best of our knowledge, this is the first of report analyzing the key regions in the C-terminal of fish herpesvirus GPCR. CaHV caused high mortality rates in fish, and this study provided valuable information and new insights into the precise interactions between herpesvirus and fish cells, and could provide new targets for antiviral agents in fish aquaculture.
